# Molar Tooth Lodged in the Tongue

**Published:** 1862-08

**Authors:** 


Molar Tooth Lodged in the Tongue.—A man about twenty years of age entered the hospital about ten days after the battle of Williamsburgh, having received a wound from a bullet which struck the right side of the lower jaw, and passed out through the upper lip. The jaw was shattered, and when he entered the hospital, there were purulent deposits connecting with the neck externally and the mouth internally. The patient was etherized, and the wound being explored, bits of bone were found everywhere buried in the substance of the cheek and the surrounding soft parts. These were extracted, and the wound healed rapidly. Some weeks afterwards, he presented himself at the hospital with a swelling in the tongue, the edge of which had been wounded by the bullet, and which until lately, he had been unable to protrude. On examination, a hard body was found imbedded in the substance of the organ, which, on being cut upon, proved to be a molar tooth which had been knocked out of the jaw and buried in the tongue.—*Boston Med. and Surg. Journal.*

